# The effects of pulmonary diseases on histologic types of lung cancer in both sexes: a population-based study in Taiwan

**DOI:** 10.1186/s12885-015-1847-z

**Published:** 2015-11-02

**Authors:** Jing-Yang Huang, Zhi-Hong Jian, Oswald Ndi Nfor, Wen-Yuan Ku, Pei-Chieh Ko, Chia-Chi Lung, Chien-Chang Ho, Hui-Hsien Pan, Chieh-Ying Huang, Yu-Chiu Liang, Yung-Po Liaw

**Affiliations:** 1 Department of Public Health and Institute of Public Health, Chung Shan Medical University, No. 110, Sec. 1 Jianguo N. Rd., Taichung City, 40201 Taiwan; 2 Department of Family and Community Medicine, Chung Shan Medical University Hospital, 40201 Taichung City, Taiwan; 3 Department of Physical Education, Fu Jen Catholic University, New Taipei City, Taiwan; 4 School of Medicine, Chung Shan Medical University, Taichung City, Taiwan; 5 Department of Pediatrics, Chung Shan Medical University Hospital, Taichung City, Taiwan; 6 Department of Chemical Technology, Songshan High School of Agriculture and Industry, Taipei City, Taiwan; 7 College of Humanities and Social Sciences, Taipei Medical University, Taipei City, Taiwan

**Keywords:** Asthma, Chronic obstructive pulmonary disease, Lung adenocarcinoma, Small cell carcinoma, Squamous cell carcinoma, Tuberculosis

## Abstract

**Background:**

The associations between pulmonary diseases (asthma, chronic obstructive pulmonary disease [COPD], and tuberculosis [TB]) and subsequent lung cancer risk have been reported, but few studies have investigated the association with different histologic types of lung cancer.

**Methods:**

Patients newly diagnosed with lung cancer from 2004 to 2008 were identified from the National Health Insurance Research Database in Taiwan. Histologic types of lung cancer were further confirmed using the Taiwan Cancer Registry Database. Cox proportional hazards regression was used to calculate the hazard ratio (HR) of asthma, COPD, and TB and to estimate the risk of specific types of lung cancer.

**Results:**

During the study period, 32,759 cases of lung cancer were identified from 15,219,024 insurants aged 20 years and older. In men and women, the adjusted HR estimates of squamous cell carcinoma were respectively 1.37 (95 % confidence interval [CI], 1.21–1.54) and 2.10 (95 % CI, 1.36–3.23) for TB, 1.52 (95 % CI, 1.42–1.64) and 1.50 (95 % CI, 1.21–1.85) for asthma, and 1.66 (95 % CI, 1.56–1.76) and 1.44 (95 % CI, 1.19–1.74) for COPD. Similarly, the adjusted HR estimates of adenocarcinoma were respectively 1.33 (95 % CI, 1.19–1.50) and 1.86 (95 % CI, 1.57–2.19) for TB, 1.13 (95 % CI, 1.05–1.21) and 1.18 (95 % CI, 1.09–1.28) for asthma, and 1.50 (95 % CI, 1.42–1.59) and 1.33 (95 % CI, 1.25–1.42) for COPD. The HRs of small cell carcinoma were respectively 1.24 (95 % CI, 1.01–1.52) and 2.23 (95 % CI, 1.17–4.25) for TB, 1.51 (95 % CI, 1.35–1.69) and 1.63 (95 % CI, 1.16–2.27) for asthma, and 1.39 (95 % CI, 1.26–1.53) and 1.78 (95 % CI, 1.33–2.39) for COPD.

**Conclusions:**

Asthma, COPD, and TB were associated with an increased risk of all major subtypes of lung cancer. The risk was the highest among women with TB.

## Background

Lung cancer is the second leading diseases contributing to years of life lost because of premature mortality [1]. Among histologic types of lung cancer, adenocarcinoma is the most common subtype in Asians but not in Europeans [2, 3]. Typical risk factors for lung cancer include smoking and exposure to arsenic, chromium, radon, or air pollution [4, 5]. Smoking is the major risk factor for lung cancer, particularly squamous cell carcinoma (SqCC) [6]. However, a previous study demonstrated that most Taiwanese women with lung cancer are non-smokers [7]. A vast majority of smokers do not seem to develop lung cancer. Although smoking is a potential risk factor, other factors may also be linked to the increased risk of lung cancer.

Recent studies have concluded that chronic inflammation may be linked to lung carcinogenesis [8]. Among intrinsic pulmonary diseases, chronic obstructive pulmonary disease (COPD) [9, 10], asthma [11], and tuberculosis (TB) [12] are associated with lung cancer. Smokers with COPD have a higher risk of SqCC [13, 14]. Asthma is associated with an increased risk of SqCC and small cell carcinoma (SmCC) but is weakly associated with adenocarcinoma [15, 16]. TB is also associated with an increased risk of SqCC and adenocarcinoma but not SmCC [17]. An association between TB and lung adenocarcinoma has been reported in non-westernized countries [18]. Furthermore, the association of lung cancer with diabetes [19] and dyslipidemia [20–22] has been reported. Data on pulmonary diseases and specific histologic types of lung cancer are considerable limited in Taiwan.

For a detailed evaluation of the relationship between pulmonary diseases and histologic types of lung cancer, a population-based cohort study is highly desirable. However, few such studies have been conducted. This study assessed whether pulmonary diseases are associated with an increased risk of specific types of lung cancer.

## Methods

### Database

The National Health Insurance Research Database (NHIRD) contains enrollment files, claims data, catastrophic illness files, and treatment registries. The national health insurance program covers more than 99 % of the population of Taiwan. The NHIRD is one of the largest administrative health care databases that is broadly used in academic studies [23–25]. This study used the linked databases of the NHIRD, Taiwan Cancer Registry Database (TCRD), and National Death Registry Database (NDRD) with the permission of the Department of Statistics, Ministry of Health and Welfare of Taiwan. The source data was encrypted and the data extracted was anonymous. This study was approved by the Institutional Review Board of the Chung-Shan Medical University Hospital, Taiwan.

### Identification of Patients With Lung Cancer

In this study, 17,859,318 residents aged 20 years and older were initially enrolled. We excluded patients diagnosed with lung cancer before 2003 (*n* = 39,623) and those with incomplete information on sex (*n* = 2,600,565), registry data (*n* = 5), and death (*n* = 101). Finally, 15,219,024 patients (8,002,536 men and 7,216,488 women) were enrolled in this study. Patients newly diagnosed with lung cancer in 2004 were followed up until death, loss to follow-up, or the study end in 2008. Lung cancer was identified using the *International Classification of Diseases, Ninth Revision, Clinical Modification* (ICD-9-CM) code 162.

Furthermore, histologic types of lung cancer were confirmed using the TCRD. The registry contains data on cancer types, initial tumor stages, and histology. Lung cancer was identified using the ICD-9-CM code 162 or ICD 10 codes C34.0, C34.1, C34.2, C34.3, C34.8, and C34.9 in the TCRD. Morphological diagnoses were determined using the ninth revision of the *International Classification of Diseases for Oncology* (ICD-O) on the basis of the following ICD-O codes: 80522, 80523, 80702, 80703, 80713, 80723, 80733, 80743, 80763, 80823, 80833, and 80843 for lung SqCC; 80503, 81402, 81403, 81413, 81433, 82113, 82503, 82513, 82523, 82553, 82603, 83103, 83233, 84603, 84803, 84813, 84903, and 85003 for adenocarcinoma; 80023, 80412, 80413, 80423, 80433, 80453, and 94733 for SmCC; and 80123, 80143, 80203, 80213, 80303, and 80313 for large cell carcinoma.

The linked databases were used to retrieve information on the age of lung cancer diagnosis, follow-up time (in person-years), and survival time and to minimize potentially unconfirmed cancer diagnoses.

### Variables of Exposure

Baseline variables included age, sex, urbanization level, geographical area, low income, and comorbidities. To reduce bias, the diagnoses of pulmonary diseases and comorbidities were confirmed by more than two outpatient visits or one admission between 2001 and 2003. Pulmonary diseases and comorbidities were defined using the following ICD-9-CM codes: asthma (493), COPD (490, 491, 492, 494, and 496), TB (010 – 012, and137.0), chronic kidney disease (585 and 586), type II diabetes mellitus (250, which excludes type I diabetes mellitus), and hyperlipidemia (272). Smoking, a major risk factor for lung cancer, COPD, and other cancer types are not available in the NHIRD [26, 27]; hence, this prevented direct adjustment for all possible confounders. However, smoking -related cancers such as lip, oral cavity, nasal cavity, pharynx, larynx, esophagus (ICD-9-CM codes: 140–150 and 160–161), pancreas (ICD-9-CM code 157), kidney, and bladder cancers (ICD-9-CM codes 188 and 189) were adjusted [28].

### Statistical Analyses

All statistical analyses were conducted using the SAS statistical package (Version 9.3; SAS Institute, Inc., Cary, NC). The characteristics of the study population were compared using the chi-square test. A p value of < 0.05 was statistically significant. To evaluate the effect of age, patients were classified according to sex and age (20–39, 40–49, 50–59, 60–69, 70–79, and ≥80 years). All cities and towns were divided into three urbanization levels: low, medium, and high. The Cox proportional hazards regression model was used to estimate the hazard ratios (HRs) of histologic types of lung cancer while controlling for age, geographical area, urbanization level, low income, and comorbidities.

## Results

During the study period, 32,759 cases of lung cancer were identified. Of all patients diagnosed, 47.3 % had adenocarcinoma (M: F, 8,778: 6,712), 20.3 % had SqCC (5,877: 760), 9.2 % had SmCC (2,751: 268), 0.7 % had large cell carcinoma (183: 57), and 23.2 % had other diseases (5,283: 2,090). The demographic characteristics and comorbidities of the study population are displayed in Table 1. Patients with lung cancer had higher rates of asthma, COPD, TB, hyperlipidemia, diabetes, chronic kidney disease, and smoking-related cancer than individuals without lung cancer did.

**Table 1:** Characteristics of the Study Population

	Lung cancer (*N* = 32759)	Non-lung cancer (*N* = 15186265)	*p*-value
Lung diseases (%)			
Tuberculosis	1052 (3.2)	110469 (0.7)	< 0.001
Asthma	4380 (13.4)	747889 (4.9)	< 0.001
COPD	7883 (24.1)	1201101 (7.9)	< 0.001
Sex (%)			< 0.001
Men	22872 (69.8)	7979664 (52.6)	
Women	9887 (30.2)	7206601 (47.4)	
Age (years, %)			< 0.001
20–39	1049 (3.2)	7221512 (47.6)	
40–49	3580 (10.9)	3464916 (22.8)	
50–59	5937 (18.1)	2209547 (14.6)	
60–69	7374 (22.5)	1036090 (6.8)	
70–79	10544 (32.2)	823322 (5.4)	
≧80	4275 (13.1)	430878 (2.8)	
Low income (%)^a^	889 (2.7)	245045 (1.6)	< 0.001
Comorbidities (%)			
Diabetes	5680 (17.3)	1060714 (7.0)	< 0.001
Hyperlipidemia	5988 (18.3)	1347931 (8.9)	< 0.001
Chronic kidney disease	961 (2.9)	149730 (1.0)	< 0.001
Smoking-related cancers^b^	777 (2.4)	103201 (0.7)	< 0.001
Geographical area (%)			< 0.001
Taipei City	9236 (28.2)	4851844 (32.0)	
North	3920 (12.0)	1961790 (12.9)	
Central	6393 (19.5)	2936377 (19.3)	
South	6614 (20.2)	2366398 (15.6)	
Kaohsiung-Pingtung	5489 (16.8)	2628117 (17.3)	
East	1107 (3.3)	441739 (2.9)	
Urbanization (%)			< 0.001
High	13889 (42.4)	7519048 (49.5)	
Mid	12338 (37.7)	5575331 (36.7)	
Low	6532 (19.9)	2091866 (13.8)	
Death in 2004–2008 (%)	27718 (84.6)	648922 (4.3)	< 0.001
Follow-up time	84505	73801819	
(person-year)			
Histologic type (%)			
Squamous cell carcinoma	6637 (20.3)		
Adenocarcinoma	15490 (47.3)		
Small cell carcinoma	3019 (9.2)		
Large cell carcinoma	240 (0.7)		
Others	7373 (22.5)		

^a^Income is lower than the level required for charging premium

^b^Smoking-related cancers included lip, oral cavity, nasal cavity, pharynx, larynx, and esophagus, pancreas, kidney and bladder cancers that were prior to a diagnosis of lung cancer

Abbreviations: *COPD*, chronic obstructive pulmonary disease

In Table 2, Cox regression analysis revealed a significantly high incidence of lung cancer in male patients with COPD (HR, 1.56; 95 % confidence interval [CI], 1.51–1.61), asthma (HR, 1.36; 95 % CI, 1.30–1.41), TB (HR, 1.35; 95 % CI, 1.26–1.44), low income (HR, 1.14; 95 % CI, 1.05–1.23), hyperlipidemia (HR, 1.07; 95%CI, 1.04–1.11), and smoking-related cancer (HR, 1.79; 95 % CI, 1.68–1.90). The risk of lung cancer was high in female patients with TB (HR, 1.97; 95 % CI, 1.73–2.24), COPD (HR, 1.33; 95 % CI, 1.26–1.41), asthma (HR, 1.26; 95 % CI, 1.18–1.34), low income (HR, 1.36; 95 % CI, 1.20–1.54), hyperlipidemia (HR, 1.13; 95 % CI, 1.07–1.19), and smoking related cancer (HR, 2.28; 95 % CI, 2.02–2.57).

**Table 2:** Hazard Ratios and 95 % Confidence Intervals of Lung Cancer Stratified by Sex

	Male			Female	
	HR (95 % CI)	*P* value		HR (95 % CI)	*P* value
Lung diseases					
Tuberculosis	1.35 (1.26–1.44)	< 0.001	1.97 (1.73–2.24)		<0.001
Asthma	1.36 (1.30–1.41)	< 0.001	1.26 (1.18–1.34)		<0.001
COPD	1.56 (1.51–1.61)	< 0.001	1.33 (1.26–1.41)		<0.001
Low income^a^	1.14 (1.05–1.23)	0.001	1.36 (1.20–1.54)		< 0.001
Age group					
20–39	0.14 (0.13–0.16)	< 0.001	0.15 (0.14–0.17)		< 0.001
40–49	Reference		Reference		
50–59	2.80 (2.65–2.96)	< 0.001	2.17 (2.03–2.31)		< 0.001
60–69	7.30 (6.93–7.69)	< 0.001	4.35 (4.07–4.65)		< 0.001
70–79	12.70 (12.08–13.34)	< 0.001	6.70 (6.26–7.16)		< 0.001
≧80	8.91 (8.43–9.43)	< 0.001	5.72 (5.30–6.17)		< 0.001
Comorbidities					
Diabetes	1.00 (0.96–1.04)	0.926	1.01 (0.96–1.07)		0.689
Hyperlipidemia	1.07 (1.04–1.11)	<0.001	1.13 (1.07–1.19)		<0.001
Chronic kidney disease	0.90 (0.84–0.97)	0.004	0.87 (0.76–0.99)		0.035
Smoking–related cancers^b^	1.79 (1.68–1.90)	<0.001	2.28 (2.02–2.57)		<0.001

Adjustments were made to estimate HRs for all covariates (lung diseases, low income, age, comorbidities, urbanization and geographic area)

^a^Income is lower than the level required for charging premium

^b^Smoking-related cancers included lip, oral cavity, nasal cavity, pharynx, larynx, and esophagus, pancreas, kidney and bladder cancers that were prior to a diagnosis of lung cancer

Abbreviations: *CI*, confidence interval; *COPD*, chronic obstructive pulmonary disease; *HR*, hazard ratio

Table 3 presents the adjusted HRs for SqCC stratified by sex. The incidence of SqCC was high in male patients with COPD (HR, 1.66; 95 % CI, 1.56–1.76), asthma (HR, 1.52; 95 % CI, 1.42–1.64), TB (HR, 1.37; 95 % CI, 1.21–1.54), and smoking-related cancer (HR, 2.58; 95 % CI, 2.33–2.86). The HRs of SqCC in women with TB, asthma, COPD, and smoking-related cancer were 2.10 (95 % CI, 1.36–3.23), 1.50 (95 % CI, 1.21–1.85), 1.44 (95 % CI, 1.19–1.74), and 3.98 (95 % CI, 2.84–5.57), respectively.

**Table 3:** Hazard Ratios and 95 % Confidence Intervals of Squamous Cell Carcinoma Stratified by Sex

	Male			Female	
	HR (95 % CI)	*P* value		HR (95 % CI)	*P* value
Lung diseases					
Tuberculosis	1.37 (1.21–1.54)	< 0.001	2.10 (1.36–3.23)		<0.001
Asthma	1.52 (1.42–1.64)	< 0.001	1.50 (1.21–1.85)		<0.001
COPD	1.66 (1.56–1.76)	< 0.001	1.44 (1.19–1.74)		<0.001
Low income^a^	1.15 (0.99–1.34)	0.066	2.35 (1.66–3.32)		<0.001
Age group					
20–39	0.12 (0.09–0.15)	< 0.001	0.14 (0.09–0.20)		<0.001
40–49	Reference		Reference		
50–59	3.59 (3.16–4.08)	< 0.001	2.05 (1.62–2.59)		<0.001
60–69	12.38 (10.98–13.95)	< 0.001	4.47 (3.54–5.65)		<0.001
70–79	21.39 (19.03–24.04)	< 0.001	6.23 (4.89–7.92)		<0.001
≧80	13.36 (11.74–15.19)	< 0.001	4.66 (3.51–6.19)		<0.001
Comorbidities					
Diabetes	1.00 (0.93–1.07)	0.922	1.14 (0.94–1.39)		0.169
Hyperlipidemia	0.98 (0.92–1.06)	0.668	0.97 (0.80–1.17)		0.717
Chronic kidney disease	0.85 (0.73–0.97)	0.020	0.85 (0.53–1.34)		0.473
Smoking–related cancers^b^	2.58 (2.33–2.86)	< 0.001	3.98 (2.84–5.57)		<0.001

Adjustments were made to estimate HRs for all covariates (lung diseases, low income, age, comorbidities, urbanization and geographic area)

^a^Income is lower than the level required for charging premium

^b^Smoking-related cancers included lip, oral cavity, nasal cavity, pharynx, larynx, and esophagus, pancreas, kidney and bladder cancers that were prior to a diagnosis of lung cancer

Abbreviations: *CI*, confidence interval; *COPD*, chronic obstructive pulmonary disease; *HR*, hazard ratio

Table 4 provides the HRs of adenocarcinoma stratified by sex. The risk of adenocarcinoma was high in male patients with COPD (HR, 1.50; 95 % CI, 1.42–1.59), TB (HR, 1.33; 95 % CI, 1.19–1.50), asthma (HR, 1.13; 95 % CI, 1.05–1.21), hyperlipidemia (HR, 1.19; 95 % CI, 1.12–1.26), and smoking-related cancer (HR, 1.46; 95 % CI, 1.30–1.63). The HRs of adenocarcinoma in female patients with TB, COPD, asthma, hyperlipidemia, and smoking-related cancer were 1.86 (95 % CI, 1.57–2.19), 1.33 (95 % CI, 1.25–1.42), 1.18 (95 % CI, 1.09–1.28), 1.19 (95 % CI, 1.12–1.26), and 2.00 (95 % CI, 1.71–2.35), respectively.

**Table 4:** Hazard Ratios and 95 % Confidence Intervals of Adenocarcinoma Stratified by Sex

	Male			
	HR (95 % CI)	*P* value	HR (95 % CI)	*P* value
Lung diseases				
Tuberculosis	1.33 (1.19–1.50)	< 0.001	1.86 (1.57–2.19)	< 0.001
Asthma	1.13 (1.05–1.21)	< 0.001	1.18 (1.09–1.28)	< 0.001
COPD	1.50 (1.42–1.59)	< 0.001	1.33 (1.25–1.42)	< 0.001
Low income^a^	1.07 (0.94–1.22)	0.322	1.18 (1.00–1.39)	0.055
Age group				
20–39	0.15 (0.13–0.17)	< 0.001	0.15 (0.13–0.17)	< 0.001
40–49	Reference		Reference	
50–59	2.45 (2.27–2.64)	< 0.001	2.17 (2.01–2.34)	< 0.001
60–69	5.23 (4.85–5.63)	< 0.001	4.10 (3.80–4.44)	< 0.001
70–79	8.15 (7.59–8.76)	< 0.001	5.93 (5.46–6.43)	< 0.001
≧80	5.61 (5.15–6.11)	< 0.001	4.03 (3.64–4.45)	< 0.001
Comorbidities				
Diabetes	0.95 (0.90–1.01)	0.106	0.95 (0.89–1.02)	0.128
Hyperlipidemia	1.19 (1.12–1.26)	< 0.001	1.19 (1.12–1.26)	< 0.001
Chronic kidney disease	0.86 (0.76–0.98)	0.019	0.84 (0.71–0.99)	0.040
Smoking–related cancers^b^	1.46 (1.30–1.63)	<0.001	2.00 (1.71–2.35)	< 0.001

Adjustments were made to estimate HRs for all covariates (lung diseases, low income, age, comorbidities, urbanization and geographic area)

^a^Income is lower than the level required for charging premium

^b^Smoking-related cancers included lip, oral cavity, nasal cavity, pharynx, larynx, and esophagus, pancreas, kidney and bladder cancers that were prior to a diagnosis of lung cancer

Abbreviations: *CI*, confidence interval; *COPD*, chronic obstructive pulmonary disease; *HR*, hazard ratio

Table 5 displays the adjusted HRs of SmCC stratified by sex. The risk of SmCC was high in male patients with asthma (HR, 1.51; 95 % CI, 1.35–1.69), COPD (HR, 1.39; 95 % CI, 1.26–1.53), TB (HR, 1.24; 95 % CI, 1.01–1.52), and smoking-related cancer (HR, 1.35; 95 % CI, 1.10–1.66). The HRs of SmCC in women with TB, COPD, asthma, and smoking-related cancer were 2.23 (95 % CI, 1.17–4.25), 1.78 (95 % CI, 1.33–2.39), 1.63 (95 % CI, 1.16–2.27), and 3.71 (95 % CI, 2.12–6.49), respectively.

**Table 5:** Hazard Ratios and 95 % Confidence Intervals of Small Cell Carcinoma by Stratified by Sex

	Male		Female	
	HR (95 % CI)	*P* value	HR (95 % CI)	*P* value
Lung diseases				
Tuberculosis	1.24 (1.01–1.52)	0.037	2.23 (1.17–4.25)	0.015
Asthma	1.51 (1.35–1.69)	< 0.001	1.63 (1.16–2.27)	0.005
COPD	1.39 (1.26–1.53)	< 0.001	1.78 (1.33–2.39)	<0.001
Low income^a^	0.96 (0.75–1.23)	0.738	2.91 (1.80–4.68)	< 0.001
Age group				
20–39	0.09 (0.06–0.12)	< 0.001	0.10 (0.04–0.27)	< 0.001
40–49	Reference		Reference	
50–59	3.47 (2.93–4.10)	< 0.001	3.73 (2.31–6.01)	< 0.001
60–69	10.60 (9.04–12.43)	< 0.001	10.86 (6.80–17.33)	< 0.001
70–79	17.1 (14.64–19.98)	< 0.001	14.19 (8.73–23.07)	< 0.001
≧80	9.61 (8.03–11.49)	< 0.001	14.97 (8.97–24.97)	< 0.001
Comorbidities				
Diabetes	1.07 (0.96–1.18)	0.226	1.24 (0.92–1.69)	0.162
Hyperlipidemia	1.09 (0.98–1.21)	0.100	0.84 (0.61–1.15)	0.264
Chronic kidney disease	0.70 (0.55–0.89)	0.003	0.90 (0.44–1.84)	0.774
Smoking-related cancers^b^	1.35 (1.10–1.66)	0.004	3.71 (2.12–6.49)	< 0.001

Adjustments were made to estimate HRs for all covariates (lung diseases, low income, age, comorbidities, urbanization and geographic area)

^a^Income is lower than the level required for charging premium

^b^Smoking-related cancers included lip, oral cavity, nasal cavity, pharynx, larynx, and esophagus, pancreas, kidney and bladder cancers that were prior to a diagnosis of lung cancer

Abbreviations: *CI*, confidence interval; *COPD*, chronic obstructive pulmonary disease; *HR*, hazard ratio

## Discussion

This study demonstrated that male and female patients with TB, asthma, and COPD had increased risks of lung SqCC, adenocarcinoma, and SmCC. Determining risk factors for specific types of lung cancer can help physicians gain a detailed understanding of the etiology of lung cancer and therefore identify the high-risk population for screening. To the best of our knowledge, no study has investigated the association between pulmonary diseases and histologic types of lung cancer. According to the study results, heterogeneity was observed in the risk factors for lung cancer and the different histologic types in male and female patients.

A population-based, case-control study of female nonsmokers revealed an increased risk of lung cancer in patients with TB who were diagnosed before the age of 21 years [29]. The incidence rate ratio of lung cancer in the TB cohorts was 1.98 (95%CI, 1.37–2.83) 2 – 4 years after TB infection [12]. A hospital-based, case-control study involving interviews of 226 female nonsmokers with lung cancer and 279 controls demonstrated that TB increased the risk of lung cancer (odds ratio [OR], 4.7; 95 % CI, 1.6–13.2) [30]. Yu et al. found an increased risk of lung cancer among patients with TB (HR, 3.32; 95 % CI, 2.70–4.09), which was higher than that of COPD (HR, 2.30; 95 % CI, 2.07–2.55) [28]. A systematic review identified a direct relationship between preexisting TB and lung cancer, particularly adenocarcinoma (relative risk [RR], 1.6; 95 % CI, 1.2–2.1) [18]. A study conducted in Taiwan demonstrated that TB was an independent risk factor for SqCC, SmCC, and adenocarcinoma in men and women [6]. Such an association is particularly crucial in Taiwan, where the prevalence of TB is high [31, 32]. Compared with asthma and COPD, TB appears to have a stronger association with lung cancer among women. Additional studies are necessary to assess the possible mechanisms of this association.

In this study, COPD was associated with the risk of the major types of lung cancer. Chronic airway inflammation is a major risk factor for COPD and is also associated with an increased risk of lung cancer [13]. A study involving the 22-year follow-up of 5,402 participants concluded that moderate-to-severe obstructive pulmonary disease was associated with a higher risk of incident lung cancer (HR, 2.8; 95 % CI, 1.8–4.4) [33]. Denholm et al. found that chronic bronchitis and emphysema were positively associated with lung cancer after adjusting for other respiratory diseases and smoking (OR, 1.33; 95 % CI, 1.20–1.48 for men; OR, 1.50; 95 % CI, 1.21–1.87 for women) [34]. The prevalence of smoking is almost 10-fold higher in Taiwanese men than that in women [35]. However, the smoking status of the study population was not available. This may be the reason for the observed differences between men and women. Chronic bronchitis and emphysema increased the risk of SqCC (HR, 1.54; 95 % CI, 1.09–2.18) independent of smoking [36]. COPD also increased the risk of SqCC in smokers [13]. Pesch et al. performed a pooled analysis of case-control studies including 13,169 cases and 16,010 controls from Europe and Canada [37]. Their analysis demonstrated that adenocarcinoma was the most prevalent subtype in never-smokers and women. The ORs were elevated for exposure to cigarette smoke and were higher for SqCC and SmCC than for adenocarcinoma. Freedman et al. recruited 279,214 men and 184,623 women aged 50–71 years from eight states in the United States to evaluate whether women were more susceptible to lung cancer caused by cigarette smoking than men [38]. Their results illustrated that the HRs of adenocarcinoma, SmCC, and undifferentiated tumors were similar between men and women among ex-smokers and current smokers. However, among current smokers, the HR of SqCC in men was approximately 2-fold higher than that in women.

This study also suggests that patients with asthma are at an increased risk of three histological types (SqCC, adenocarcinoma, and SmCC) of lung cancer. Asthma is one of the most common chronic airway diseases and affects 300 million people of all ages and ethnicities [39]. In Taiwan, the prevalence of asthma has increased to 11.9 % [40]. Because asthma causes complex chronic airway inflammation, it has been hypothesized to lead to carcinogenesis [8]. Case-control studies have produced varied results for the association between asthma and lung cancer [41, 42]. In a Swedish cohort with a hospital-discharge diagnosis of asthma, the standardized incidence rate ratio of lung cancer was 1.51 in men (95 % CI, 1.38–1.65) and 1.78 in women (95 % CI, 1.55–2.03), and the risk of histologic types of lung cancer was higher in patients with SqCC and SmCC [16]. In a meta-analysis, the RRs were 1.69 (95 % CI, 1.26–2.26) for SqCC, 1.71 (95 % CI, 0.99–2.95) for SmCC, and 1.09 (95 % CI, 0.88–1.36) for adenocarcinoma [15].

In this study, hyperlipidemia was also associated with an increased risk of adenocarcinoma. Hyperlipidemia is a component of metabolic syndrome and is associated with insulin resistance [43]. Hyperinsulinemia, hyperglycemia, and chronic inflammation play a vital role in the neoplastic process [44]. High serum triglyceride concentrations are associated with an increased risk of lung cancer (fourth vs first quartile: HR, 1.94; 95 % CI, 1.47–2.54) [22]. Additional studies are required to assess the association between hyperlipidemia and adenocarcinoma.

Evaluating the temporal relationship between pulmonary diseases and subsequent lung cancer in case-control studies is difficult. Previous studies might have yielded inconclusive results because they focused mainly on the high-risk populations of heavy smokers. This study has several strengths. First, our data were retrieved from combined databases (NHIRD, TCRD and NDRD) that included all residents; hence, recall and selection bias was minimized. Second, the histologic type of lung cancer was confirmed using the TCRD. Nevertheless, our study has some limitations. First, the NHIRD does not contain detailed clinical data and information on lifestyle-related factors such as smoking, obesity, physical inactivity, dietary habits, and family history, which are closely associated with lung cancer. Smoking is a major confounding factor of lung cancer. Biases were minimized by adjusting for COPD and smoking-related cancer. Second, patients with asthma, COPD, and TB may have used medications that may have complicated their conditions. This study did not evaluate the effects of drugs.

## Conclusions

This study demonstrated that asthma, COPD, and TB were associated with increased risks of all major subtypes of lung cancer. The risk was the highest among women with TB.

## Abbreviations

CI: Confidence interval
COPD: Chronic obstructive pulmonary disease
HR: Hazard ratio
ICD-9-CM: International Classification of Diseases, Ninth Revision, Clinical Modification code
ICD-O: International Classification of Diseases for Oncology
NDRD: National Death Registry Database
NHIRD: National Health Insurance Research Database
OR: Odds ratio
RR: Relative risk
SmCC: Small cell carcinoma
SqCC: Squamous cell carcinoma
TB: Pulmonary tuberculosis
TCRD: Taiwan Cancer Registry Database
